# The Influence of Particle Size and Calcium Content on Performance Characteristics of Metakaolin- and Fly-Ash-Based Geopolymer Gels

**DOI:** 10.3390/gels10100639

**Published:** 2024-10-07

**Authors:** Yefan Li, Yanhui Dong, Mohamed R. El-Naggar, Fucheng Wang, Yixin Zhao

**Affiliations:** 1 School of Energy and Mining Engineering, China University of Mining and Technology (Beijing), Beijing 100083, China; lyf2495076637@163.com (Y.L.); w_fuchen@163.com (F.W.); 2 Key Laboratory of Shale Gas and Geoengineering, Institute of Geology and Geophysics, Chinese Academy of Sciences, Beijing 100029, China; 3 Innovation Academy for Earth Science, Chinese Academy of Sciences, Beijing 100029, China; 4 College of Earth and Planetary Science, University of Chinese Academy of Sciences, Beijing 100049, China; 5 Radioactive Waste Management Department, Hot Laboratories and Waste Management Center, Egyptian Atomic Energy Authority, Cairo 13759, Egypt; elnaggar74@yahoo.com

**Keywords:** geopolymer gels, particle size, calcium content, compressive strength, leaching behavior, pore structure, metakaolin, fly ash

## Abstract

This research systematically investigates the influence of raw material particle size and calcium content on the geopolymerization process to gain insight into the physical and mechanical properties of geopolymer gels, including setting time, fluidity, pore structure, compressive strength, and leaching characteristics of encapsulated Cr^3+^ heavy metal ions. Utilizing a diverse range of particle sizes of metakaolin (MK; 3.75, 7.5, and 12 µm) and fly ash (FA; 18, 45, and 75 µm), along with varied calcium levels, this study assesses the dual impact of these factors on the final properties of both metakaolin- and fly-ash-based geopolymers. Employing sophisticated analytical techniques such as Scanning Electron Microscopy (SEM), Fourier Transform Infrared Spectroscopy (FTIR), and Nuclear Magnetic Resonance (NMR), the research meticulously documents alterations in chemical bonding, micro-morphology, and pore structures. Key findings reveal that reducing the size of MK and FA particles to 3.75 and 18 µm, respectively, enhances the compressive strength of their matrices by 128.37 and 297.58%, respectively, compared to their original values (63.59 and 33.87 MPa, respectively) at larger particle sizes. While smaller particle sizes significantly bolster compressive strength, they adversely affect slurry flow and reduce the leaching rates of Cr^3+^ from MK- and FA-based matrices, reaching 0.42 and 0.75 mg/L at 3.75 and 18 µm, respectively. Conversely, increased calcium content markedly enhances setting times and contributes to the formation of dense microstructures through the production of calcium aluminate silicate hydrate (C-A-S-H) gels, thus improving the overall curing performance and durability of the materials. These insights underline the importance of fine-tuning particle size and calcium content to optimize geopolymer formulations, offering substantial benefits for varied engineering applications and promoting more sustainable construction practices.

## 1. Introduction

Stabilization/solidification technology is the primary method for treating solid wastes containing heavy metals [1,2,3]. Traditional cementitious materials have limitations due to their poor performance and environmental concerns. Geopolymer (GP) gels, recognized for their effective immobilization capabilities, economic benefits, and universal applicability, offer a practical solution for long-term waste management [4]. These novel inorganic materials are synthesized from aluminosilicates and alkaline or acidic solutions where the former may be at or near ambient temperatures, making them an environmentally friendly alternative to conventional binders [5,6,7], while the latter may utilize a phosphoric acid solution with limited application due to the higher costs and environmental impacts [8].

A wide range of aluminosilicates like MK, FA, feldspar, red mud, etc., could be considered as precursors for GP synthesis [6,9,10,11]. GP gels are increasingly favored for their ease of synthesis, low environmental impact, and versatile properties, making them ideal for various applications including construction, refractory ceramics, and composites [12]. Additionally, geopolymeric matrices can be applied for immobilizing toxic wastes [13]. The performance of GP gels is influenced by factors such as the formulation of the gelling powder, the amount of alkaline activator used, and the specific treatment conditions [14,15]. The interaction between the raw material and the activator is critical, with the particle size of the raw material being a key determinant of reactivity [16]. Studies have demonstrated that the fineness of raw materials can significantly enhance the mechanical properties of GP gels [17]. El-Naggar and El-Dessouky [18] investigated the influence of four particle size fractions of MK ranging between < 38 and > 125 µm on the mechanical properties of developed GP formulations. They found that the mechanical properties were improved via four consequential stages by lowering the MK particle size to less than 38 µm, loading of 3% undissolved waste glass, loading of 7% fused waste glass at the Si-modulus of 1.55, and early age curing at 40 °C, recording 80.75, 82.36, 93.08, and 97.41 MPa, respectively [18]. However, the high viscosity of water glass solutions used in GP formulations can impair flow properties, necessitating adjustments to the liquid-solid ratio to meet construction requirements while potentially introducing some negative effects. Notably, the particle size impacts not only the flowability but also the mechanical properties and pore structure of the geopolymer gels, underscoring its importance in the overall performance of these materials [19]. The influence of particle size on geopolymer performance is not yet fully understood, and further investigation is needed to better understand this effect in both MK-based and FA-based GP gels.

Aluminosilicate cementitious powders are categorized into two groups: (1) low-calcium systems with less than 10% calcium content, such as FA (category F), which primarily provides silicon and aluminum (Si + Al) as reactive binding agents, and (2) high-calcium systems with more than 10% calcium, where silicon and calcium (Si + Ca) contribute to forming calcium aluminum silicate hydrate (C-A-S-H) networks. These networks solidify quickly and can harden at ambient temperature [20]. The growth of the electric power industry has led to increased emissions and the accumulation of high-calcium waste, necessitating effective disposal and utilization strategies for silica–aluminum materials [21]. Additionally, China produces blast furnace slag (BFS) at a rate of 100 million tons/year, while the recycling of this industrial byproduct may only reach 30–40%, causing environmental threats [22]. The usage of BFS in the production of geopolymers is a better choice to save the environment.

Thus, the present study was designed to utilize MK and FA binders with different particle sizes to prepare GP gels along with the incorporation of BFS to adjust the calcium contents. This research aimed to evaluate the impact of raw material particle size and calcium content on the pore structure, mechanical properties, and curing capabilities of the GPs. Advanced characterization techniques such as Scanning Electron Microscopy (SEM), Fourier Transform Infrared Spectroscopy (FTIR), and Low-Field Nuclear Magnetic Resonance (LF-NMR) were employed to assess changes in chemical bonding morphology, micro-morphology, and curing capacity before and after activation of the matrix components. These analyses helped to understand the effects of varying calcium content and raw material particle size on the performance of geopolymer gels made from the two types of raw materials.

## 2. Results and Discussion

### 2.1. Setting Time and Fluidity

**Figure 1:**
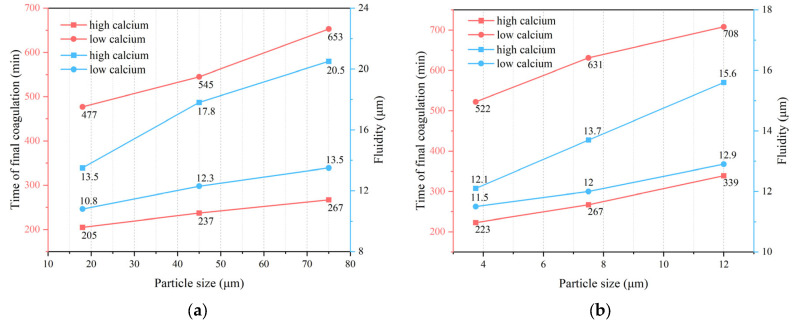
Setting time and fluidity of (**a**) fly-ash- and (**b**) metakaolin-based geopolymeric gels.

Setting time and fluidity are critical indicators of the usability of ground GP gel in engineering applications. Data demonstrated a clear trend where larger raw material particle sizes significantly prolong the setting times. Figure 1 illustrates that, in low-calcium FA-based GPs, increasing the particle size from 18 μm to 75 μm extended the final setting time from 477 to 653 min. This fast final setting of specimens, compared to what was recorded by Sayenko and co-workers [23], reflects the better performance of the examined gels for building applications. Similarly, for low-calcium MK-based GPs, the final setting time was increased from 522 to 708 min as the particle size grew from 3.5 μm to 12 μm.

Moreover, larger particle sizes result in a decrease in water bound to reactants and a relative increase in free water. This excess free water can interfere with and retard the polycondensation reactions, further prolonging the overall curing time. These results underline the importance of particle size regulation in the preparation of GPs, as precise control over particle sizes can effectively tailor the setting times to meet specific engineering requirements and performance specifications [24].

The incorporation of BFS notably reduces the setting time of gels. This phenomenon is primarily due to the slag’s content of Ca^2+^ ions, which increase the system’s calcium concentration. There is a well-documented correlation between the content of Ca^2+^ ions and setting times in alkali-activated cementitious systems; lower Ca^2+^ levels generally lead to longer initial and final setting times. Additionally, increasing the slag content accelerates the dissolution of Ca^2+^ ions, hastening the formation of polycondensation products within the gel and thus reducing the overall setting time.

Furthermore, the flowability of the GP gel improves with increased calcium content, showcasing a marked enhancement in flowability values. This improvement is largely attributed to the addition of BFS, which typically has a smaller specific surface area. As the specific surface area decreases, fluidity increases rapidly. Concurrently, the rise in calcium content reduces the proportion of other components in the mortar, and the rapid dissolution of Ca^2+^ decreases the overall solid phase, thereby increasing the gel’s fluidity.

### 2.2. Morphology from Scanning Electron Microscopy (SEM)

**Figure 2:**
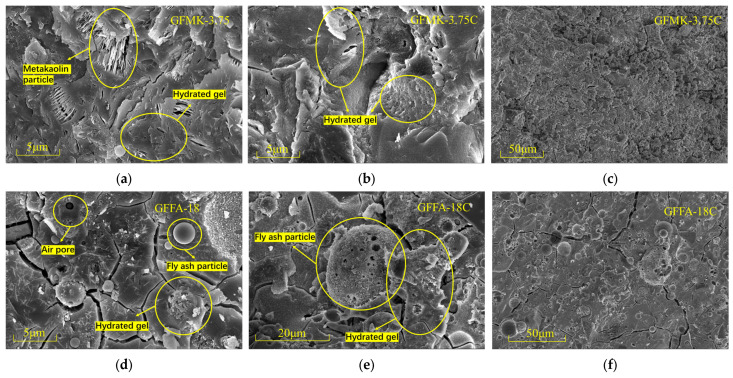
SEM images of geopolymers with different calcium contents: (**a**) GFMK-3.75; (**b**,**c**) GFMK-3.75C; (**d**) GFFA-18; (**e**,**f**) GFFA-18C. GFFA(MK)-n(C): FA represents fly ash, MK represents metakaolin, n represents particle size (μm), C represents high calcium content of 20%.

SEM tests were conducted on GP samples cured for 28 days across varying calcium content systems, with the findings illustrated in Figure 2. Figure 2a,b display the microscopic morphology of the MK-based GP in low-calcium and high-calcium systems, respectively, characterized mainly by lamellar MK feedstock and hydrated gel structures. A comparison between the two images shows a significant structural difference in the GPs when subjected to different calcium content systems. Notably, the high-calcium system’s GP features a dense gel body structure with numerous adsorbed particles at the same magnification.

When the magnification is reduced for observation in Figure 2c, it becomes apparent that the surface of the GP in the high-calcium system is covered with a substantial amount of dense gel, indicative of C-A-S-H or N-A-S-H formation. This also points to the dissolution of the glassy phase in an alkaline solution.

Figure 2d–f focus on the morphology of FA-based geopolymers. The structure predominantly comprises spherical FA particles enveloped by a calcium-containing hydrated gel, similar to the MK-based systems in the high-calcium environment as shown in Figure 2e. Further observation in Figure 2f reveals that the spherical FA particles are tightly bonded to the external gel, forming a transition layer between the feedstock and the base phase. This indicates that a geopolymerization reaction has occurred, resulting in the formation of [-Al-O-Si-] chemical bonds.

Despite being oven-cured, the GP samples still contained unreacted feedstock particles, a testament to the limited hydration process. This slow reactivity contributes to gradual strength development over the curing period, aligning with the trends observed in the compressive strength tests.

### 2.3. X-ray Diffraction (XRD) and Fourier Transform Infrared Spectroscopy (FT-IR)

**Figure 3:**
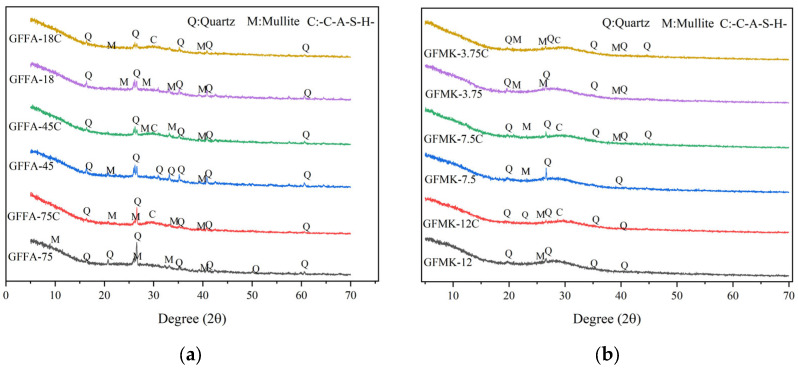
X-ray diffractometry patterns of different geopolymers: (**a**) fly-ash-based geopolymer; (**b**) metakaolin-based geopolymer. GFFA(MK)-n(C): FA represents fly ash, MK represents metakaolin, n represents particle size (μm), C represents high calcium content of 20%.

The XRD diffractograms of the samples, depicted in Figure 3, primarily show quartz and mullite as the main constituents in both FA-based and MK-based GPs, originating from the feedstock. Observations of samples with varied feedstock particle sizes indicate a reduction in the number of quartz peaks and the intensity of their diffraction signals as particle size decreases. This suggests that the quartz component, to a significant extent, has dissolved and transformed into amorphous products, implying that smaller particles favor the geopolymerization process.

In diffractograms of GPs prepared with different calcium contents, despite the high CaO content in the slag-based raw materials, no crystal peak of CaO is evident in the synthesized specimens. Instead, there is a distinct diffuse diffraction peak between 2θ = 29.5° and 35.6°, suggesting the formation of a new substance. According to Zhang and colleagues, this broad peak is identified as hydrated calcium alumino-silicate (C-A-S-H) gel. Overall, the physical phase changes in the specimens are minimal, predominantly displaying quartz and mullite. However, a new crystalline diffraction peak containing Ca^2+^ has emerged. This new peak is attributed to the release of Ca^2+^ ions, which contributes to a more balanced charge distribution within the system. Moreover, the presence of Ca^2+^ ions is known to accelerate the volcanic ash reactions in fly ash and enhance the overall degree of geopolymerization [19].

**Figure 4:**
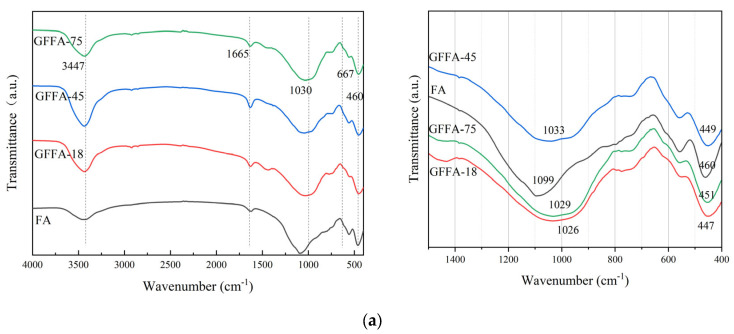

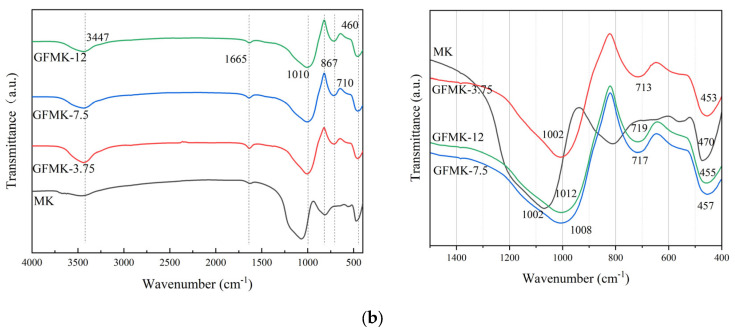
FT-IR spectra of different geopolymers: (**a**) fly-ash-based geopolymer; (**b**) metakaolin-based geopolymer. GFFA(MK)-n(C): FA represents fly ash, MK represents metakaolin, n represents particle size (μm), C represents high calcium content of 20%.

Figure 4 presents the FTIR spectra of FA-based and MK-based GPs. The broad band at 3458 cm^−1^ and the weak peak at 1657 cm^−1^ in the spectra are attributed to the O-H bond stretching and H-O-H bending vibrations of water molecules adsorbed in the interlayers, respectively. These variations in peak intensity suggest the presence of structural water in the FA geopolymers, with the band near 1657 cm^−1^ indicating water molecules enclosed within the aluminosilicate structure. Additionally, the small band at approximately 2360 cm^−1^ is identified as the IR band position of H-CO_3_.

Metakaolin exhibits distinct chemical bonding features in its pristine state. Notably, a broad vibrational peak at 1087 cm^−1^ signifies the telescopic vibrational activity of silicon–oxygen (Si-O) bonds, while the peak at 767 cm^−1^ reflects the vibrational mode of aluminum–oxygen (Al-O) bonds in the aluminum–oxygen tetrahedra. Upon transformation into GP, the characteristic Si-O and Al-O peaks initially associated with metakaolin vanish from the spectrogram. In their place, a new signature peak emerges around 1089 cm^−1^, indicative of the Si-O-T bond formed by silica–oxygen with another element, T (potentially silicon or aluminum), in the GP stereo network structure. This peak is a distinctive signature of the silicate glass phase.

The evolution of FTIR spectra from the raw material to the final product confirms that the original Al-O and Si-O bonds in metakaolin undergo disruption during the reaction process and are reorganized into new three-dimensional structural units during the polycondensation reaction. According to Hui Peng et al. [25], this structural transformation results from the partial replacement of silica–oxygen tetrahedra by aluminum–oxygen tetrahedra. Additionally, El-Naggar and Amin [26] have noted that a decrease in the strength of Si-O-T bonds, indicating a more disordered structure, is associated with a shift towards higher wave numbers, suggesting that the Si atoms are extensively replaced by Al atoms. The main characteristic bands appearing at 1031–1015 cm^−1^, due to asymmetric stretching vibrations of Si-O-T, shift depending on the degree of Si substitution by Al atoms. Higher substitution of Si leads to an aluminum-rich GP, with main bands shifting to lower wave numbers. From Figure 4, it is evident that as the particle size of the raw material decreases, the peaks of the Si-O-T bands shift to lower wave numbers, indicating that a reduction in particle size enhances the formation of an aluminum-rich GP, which favors the geopolymerization reaction.

### 2.4. Pore Structure Analysis from Low-Field Nuclear Magnetic Resonance (LF-NMR)

**Figure 5:**
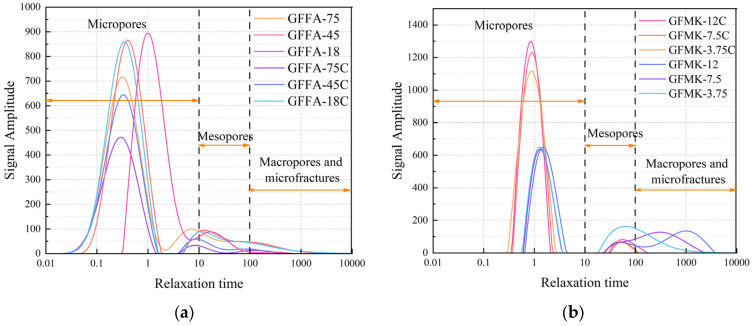
NMR *T*_2_ spectra of different geopolymers: (**a**) fly-ash-base geopolymer; (**b**) metakaolin-based geopolymer. GFFA(MK)-n(C): FA represents fly ash, MK represents metakaolin, n represents particle size (μm), C represents high calcium content of 20%.

Figure 5 showcases the pore structure characteristics of the GP as detected using LF-NMR. The *T*_2_ spectrum of the geopolymer features two distinct wave peaks, representative of microporous and mesoporous segments. The microporous segment is more pronounced than the mesoporous one, indicating a predominance of micropores, followed by mesopores, with a significantly lower presence of macropores and microfractures.

As the particle size of the raw material increases, there is a notable rise in the amplitude of the microporous waveform segment, suggesting an increase in the number of micropores corresponding to the larger particle size. Among the experimental groups of FA-based GPs, significant variations in particle size lead to noticeable differences in the wave peaks of the microporous segments in their *T*_2_ spectra. In contrast, the MK-based GPs, characterized by smaller variations in particle size among their experimental groups, show less discrepancy in their wave peaks. However, the relationship between the change in the number of pores and the size of the raw material particles remains consistent across both types of GPs.

The development of late strength in ground GPs is largely dependent on their microscopic pore structure. The inconsistency in pore size distribution plays a crucial role in contributing to variations in strength, highlighting the importance of controlling particle size to achieve desired material properties.

As the calcium content in the slurry increases, there is a noticeable decrease in the amplitude of the microporous wave segment, suggesting a reduction in the number of micropores. This trend implies that higher calcium systems may foster greater microdensification within the GP matrix due to the larger ionic radius and stronger binding ability compared to sodium ions. This densification is likely due to the rapid early hydration reactions facilitated by slag, which produces calcium aluminum silicate hydrate (C-A-S-H) gels when slag is dosed appropriately in high-calcium systems [27]. At this stage, FA serves dual roles through the “volcanic ash reaction” and the “microaggregate effect”, both of which significantly impact the material’s properties [28,29].

Conversely, MK-based GPs exhibit an opposite trend where the amplitude of the *T*_2_ spectrum peak increases with rising calcium content, indicating an increase in pore number and size. In high-calcium environments, the MK-based GP system undergoes both the alkali activation reaction from the slag and the native alkali excitation of the MK. Notably, the abundant Ca^2+^ from the slag readily combines with OH^−^ ions to form a Ca(OH)_2_ film. This film, particularly due to the lamellar particle structure of biotite, deposits on the surface of raw material particles, impeding the subsequent hydration of both the biotite and the slag. This deposition can negatively impact the overall densification and strength properties of the final material, suggesting a complex interplay of reactions that affect the structural integrity and performance of the geopolymer.

### 2.5. Influence of Particle Size and Calcium Content on Compressive Strengths

**Figure 6:**
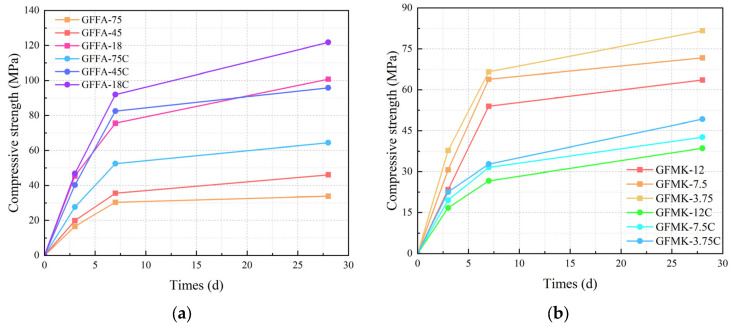
Progress of compressive strength of geopolymers with time: (**a**) fly-ash-based geopolymer; (**b**) metakaolin-based geopolymer. GFFA(MK)-n(C): FA represents fly ash, MK represents metakaolin, n represents particle size (μm), C represents high calcium content of 20%.

Figure 6 illustrates the relationship between the mechanical properties of GPs and the particle size of the raw materials used. There is a clear trend showing that as the particle size decreases, the compressive strength of the GP generally increases. For instance, the GP prepared from 75 μm fly ash displays the lowest compressive strength, achieving only 33.87 MPa after 28 days of curing. This lower strength is attributed to the relatively coarse silicon–aluminum materials in the FA, which are more difficult to activate.

In comparison, the GP made from 45 μm FA initially matches the strength of the 75 μm sample after 3 days of curing, but it shows a moderate increase after 28 days, reaching strengths of 33.87 MPa and 46.13 MPa, respectively. Remarkably, the GP derived from 18 μm fly ash significantly outperforms the others, attaining a compressive strength of 100.79 MPa.

Similarly, GPs made from MK follow the same trend as those made from FA. The compressive strength also increases with finer particle sizes. For example, a GP made from 12 μm metakaolin shows a compressive strength of 63.59 MPa after 28 days, whereas one made from 3.75 μm MK reaches a higher strength of 81.63 MPa. This pattern reaffirms that smaller particle sizes not only facilitate better activation of the materials but also contribute to significantly higher final compressive strengths.

Figure 6 also demonstrates how the compressive strength of GPs varies in systems with different calcium contents. The addition of slag significantly increases the compressive strength of the FA-based GP, with the high-calcium system achieving a maximum compressive strength of 121.77 MPa. In alkaline environments, the early hydration reaction of slag is expedited, leading to a substantial formation of calcium aluminum silicate hydrate (C-A-S-H) gel. Simultaneously, the alkaline activation also catalyzes the hydration reaction of MK, forming sodium aluminum silicate hydrate (N-A-S-H) gel. These gels intermingle and densely populate the system’s pores, creating a highly compact microstructure that boosts the material’s overall performance, including strength and durability. This enhancement aligns with earlier analyses of changes in the microporous structure and is further augmented by the “pozzolanic effect” and the “micro-aggregate effect” of FA, which improve the material’s density and, consequently, its compressive strength.

Conversely, the compressive strength of MK-based GPs in high-calcium systems exhibits a notable decrease compared to that in low-calcium systems. After 28 days, the highest strength recorded for high-calcium MK-based GPs was only 49.24 MPa, which is significantly lower than that for MK-based GPs made from low-calcium systems. This decrease in strength can be attributed to the distinct flaky structure of MK, unlike the spherical structure of fly ash. In high-calcium environments, the geopolymerization encompasses both the chemical reactions of alkaline-activated slag and MK. The abundant Ca^2+^ ions in the slag quickly bind with OH^⁻^ ions to form Ca(OH)_2_ precipitate, which then deposits and covers the surfaces of the raw material particles, creating a barrier that hampers further hydration reactions. This effect limits the depolymerization capability of the aluminosilicate in the slurry and increases the coordination number of the calcium–oxygen polyhedra, thereby enlarging the distance between the [SiO_4_] and [AlO_4_] tetrahedral monomers. This spatial increase inhibits the polymerization process to some extent, decreases the degree of polymerization, and diminishes the stability of the product structure, ultimately reducing the compressive strength.

### 2.6. Influence of Particle Size and Calcium Content on Cr^3+^ Leaching Behavior

**Figure 7:**
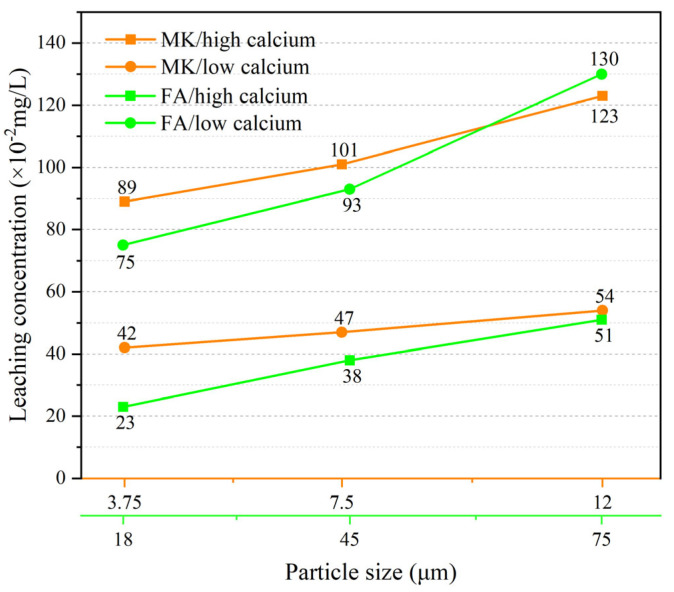
Leaching concentrations (mg/L) of chromium ions from low- and high-calcium-content fly-ash- and metakaolin-based geopolymers as affected by the particle size of base binders.

Figure 7 highlights the relationship between the leaching rates of Cr^3+^ from GPs as affected by the particle size of raw materials, revealing a clear trend: the finer the particle size, the higher the leaching rate. Specifically, as particle size increases from 18 μm to 45 μm and further to 75 μm, the leaching rate increases from 0.75 mg/L to 0.95 mg/L, and ultimately to 1.3 mg/L after 28 days of curing. This observation suggests that smaller raw material particle sizes result in smaller and denser pores within the geopolymer structure, making it more challenging for heavy metals to leach out. Additionally, the chemical bonding is tighter in GPs formed from finer particles, further reducing the leaching potential. Consequently, GPs produced from 18 μm FA demonstrate the most favorable leaching rates and compressive strength, leading to the selection of 18 μm FA for future experiments.

The trend in leaching rates is similarly consistent in MK-based GPs, where coarser particle sizes correlate with higher leaching rates. As the particle size increases from 3.75 μm to 7.5 μm and then to 12 μm, leaching rates rise from 0.42 mg/L to 0.47 mg/L, and finally to 0.54 mg/L after 28 days. The GP produced from 3.75 μm MK not only shows the best leaching rates but also superior compressive strength, making it the preferred choice for subsequent experiments involving MK-based GPs. This comprehensive analysis underlines the significant impact of particle size on both the mechanical strength and environmental performance of GPs, guiding the optimization of raw material selection to enhance the overall efficacy and sustainability of GP applications.

Figure 7 further elucidates the impact of varying calcium content on the leaching concentration of Cr^3+^. Notably, the introduction of BFS into the FA-based GP composition leads to a decreased leaching concentration of this heavy metal ion. This reduction is largely due to the formation of significant quantities of calcium aluminum silicate hydrate (C-A-S-H) gel and sodium aluminum silicate hydrate (N-A-S-H) gel in an alkaline environment, which enhances the overall performance of the material. These findings align with previous analyses indicating that the dual effects of FA—specifically the “pozzolanic effect” and the “micro-aggregate effect”—improve the material’s density, thus enhancing compressive strength and increasing the immobilization of heavy metal ions, which in turn reduces their leaching concentration.

In high-calcium systems, the leaching concentration of ions from MK-based GPs after 28 days registers at 0.53 mg/L. This is consistent with the observed changes in compressive strength, where the abundance of Ca^2+^ ions from the slag reacts with OH^−^ ions to form Ca(OH)_2_ precipitates. This reaction not only disrupts the polymerization process but also destabilizes the product structure, leading to increased ion leaching concentrations. SEM microstructural images corroborate these findings, showing an elevated leaching rate of ions. These observations suggest that while the calcium gel formed by calcium ions boosts the density of the material, it also encapsulates and immobilizes heavy metals, effectively solidifying them within the GP matrix.

## 3. Conclusions

Through the above results and discussion, one can find that the particle size and calcium content of metakaolin and fly ash significantly influence the setting time, fluidity, pore structure, and curing properties of their corresponding geopolymers. The achieved results can be summarized as follows:

1. The smaller particles of both binders with higher calcium content slowed down the setting time of geopolymers and deteriorated the flow of the slurries, suggesting a trade-off between achieving desired setting times and maintaining optimal flow properties.
2. There is a negative correlation between the raw material particle size and compressive strength, but a positive correlation with the leaching rate.
3. An increase in reactive calcium ions led to a more complex geopolymerization reaction. Not only does the geopolymerization occur, but the Ca^2+^ ions also form a calcium-containing hydrated gel. This gel interweaves with the geopolymer gel, filling the microstructure more compactly, which enhances the curing performance by creating a denser pore structure.
4. The differences between fly ash and metakaolin in their physicochemical properties exhibited different behaviors in systems with high calcium content. These variations influence how each material reacts within the geopolymer matrix, impacting the final material characteristics.

## 4. Materials and Methods

### 4.1. Materials

Different sizes of MK particles (3.75, 7.5, and 12 µm), FA particels (18, 45, and 75 µm), GGBFS, and industrial water glass (Na_2_SiO_3_) were purchased from Henan Borun Foundry Materials Co., Ltd, Zhengzhou, China. The adjustment of the silicon modulus ($\frac{SiO_{2}}{{Na}_{2}O}$) of water glass was achieved using pure-grade (≥96.0%) NaOH, a product of Shanghai Aladdin Biochemical Science and Technology Co., Ltd, Shanghai, China. (via https://www.aladdin-e.com, accessed on 20 August 2024). An analytically pure grade of chromium nitrate (Cr(NO_3_)_3_.9H_2_O) (Shanghai Aladdin Biochemical Science and Technology Co. Ltd, Shanghai, China. via https://www.aladdin-e.com, accessed on 20 August 2024) was selected to investigate the leachability of heavy metals under the examined curing conditions. The laboratory’s own deionized water was applied during all experiments. Table 1 shows the elemental analysis of the materials used in the study.

### 4.2. Synthesis of Geopolymeric Formulations

An experimental design was constructed (Table 2) to investigate the effect of particle size of the base binders (MK and FA) on pore structure and the subsequent mechanical properties and curing capacities of low- and high-calcium-content formulations. The low-calcium MK- and FA-based reactive single-component systems (CaO = 0.5 and 6.42%, respectively) were synthesized using MK and FA as base binders. Additionally, high-calcium MK- and FA-based binary-component reactive systems were synthesized, and GGBFS was applied to adjust the calcium oxide ratio to 20%. For all systems, the particle sizes of the raw MK binder were 3.75, 7.5, and 12 µm, while those of the raw FA binder were 18, 45, and 75 µm. The Si-modulus of the applied alkaline activator solution (AAS) was adjusted to 1.5 using granular sodium hydroxide. According to the pre-determined experimental ratios, the activator-to-binder ratio (L/S) was adjusted to 0.7 and 1.6 for FA- and MK-based formulations, respectively. The AAS was added to the mixtures according to the prescribed L/S, and the mixtures were transferred to the cement cleaner mixer. The container of the mixer was positioned on the mixing stand and lifted to the mixing working position, and the mixing equipment was started, firstly at a low speed to ensure that all the ingredients were homogeneously mixed; this stage lasted for 10 min. After mixing, the slurry attached to the blades and the walls of the pot was scraped back into the central part of the pot. The well-mixed slurries were injected into polypropylene molds with dimensions of 40 × 40 × 40 mm^3^ and Φ25 × 40 mm^3^, respectively, the former to examine their mechanical properties and the latter to observe their microscopic pore structure. The molds were then vibrated and compacted to expel internal air bubbles, and then a scraper was applied to flatten the surface of the gel and scrape away the excess gel to ensure a smooth surface. Surfaces were covered with a layer of cling film to prevent evaporation of water. Finally, the molds were placed in a 40 °C oven for 4 h and then left for a curing time of 28 days [30].

### 4.3. Characterizations

#### 4.3.1. Accessibility of Gels

Following the national standard of China, ‘Test Methods for Water Requirement of Normal Consistency, Setting Time, and Soundness of Portland Cement’ (GB/T 1346-2011 [31]), the prepared GP gel was injected into designated test molds to accurately measure the setting time using a Vickers meter. The flowability was assessed in accordance with the standardized methods for testing the uniformity of concrete admixtures (GB/T 8077-2012 [32]).

#### 4.3.2. Microstructural Characteristics

A Fourier transform infrared spectrometer (Bruker VERTEX 70V, Billerica, MA, USA) was employed to analyze atomic bonding within the wave number range of 400–4000 cm^−1^, facilitating insights into the molecular interactions. The oxide composition of the raw materials (MK, FA, and GGBFS) was determined using X-ray fluorescence (PANalytical X′Pert PRO, Almelo, The Netherlands), while the physical phase identification of the samples was conducted using an X-ray diffractometer (PANalytical X′Pert PRO, Almelo, The Netherlands). The diffraction angle (2θ) ranged from 5 to 70° with the scanning speed of 0.01°/s and the step width of 0.01°. MDI Jade 6 software was utilized for analyzing XRD spectra and comparing them with the Crystallography Open Database. Additionally, Scanning Electron Microscopy (FEI Nova Nano SEM450, Hillsboro, OR, USA) was utilized to characterize the apparent pore structure of the samples, offering detailed visualization and analysis of the microstructural features.

The concentration of Cr^3+^ was obtained by inductively coupled plasma mass spectrometry (PerkinElmer SCIEX ELAN DRC-e, Waltham, MA, USA) measurements.

#### 4.3.3. Pore Structure

The pore structure of the samples was characterized using Nuclear Magnetic Resonance (NMR) analysis, in which the geopolymer solid material to be tested was saturated and filled with a liquid (generally a wettable liquid, water was used in this experiment).

In order to fully describe the transverse relaxation time *T*_2_ of a fluid in a porous medium, it can be expressed with the help of a mathematical model [33]:

(1) $$\frac{1}{T_{2}}=\frac{1}{T_{2B}}+\frac{1}{T_{2S}}+\frac{1}{T_{2D}}=\frac{1}{T_{2B}}+\rho \frac{S}{V}+\frac{{D_{m}\left(GT_{E}\gamma \right)}^{2}}{12}$$

where *T*_2B_ is the free relaxation time; *T*_2S_ is the surface relaxation time; *T*_2D_ is the diffusion relaxation time; *ρ* is the transverse surface relaxation strength; *S* is the pore surface area; *V* is the pore volume; *D*_m_ is the molecular diffusion coefficient; *G* is the magnetic field gradient; *T*_E_ is the echo time; *γ* is the ratio of magnetic spins.

The transverse relaxation time (*T*_2_) of the geopolymer samples was measured using the Carr–Purcell–Meiboom–Gill (CPMG) pulse sequence. For these measurements, the following parameters were set: a waiting time of 2000 ms, an echo time of 0.2 ms, 8000 echoes, and 4 sampling repetitions.

## Figures and Tables

**Table 1:** Quantitative elemental analysis of solid binders and the utilized water glass.

(wt.%)	SiO_2_	Al_2_O_3_	Fe_2_O_3_	Cl	CaO	SO_3_	H_2_O	OH^−^	Na_2_O	LOI
Fly ash	46.6	38.2	3.2	0.015	4.2	2.1	0.85	1.2		2.8
Metakaolin	53.00	41.50	0.80							
GGBFS	33.58	15.22	0.62		33.41					
Water glass	31.00						57.45		11.55	

**Table 2:** Experimental group design of low- and high-calcium-content MK- and FA-based formulations.

Number	Raw Materials	Particle Size (μm)	Calcium Content (wt.%)	Heavy Metal Content
GFMK-12	MK	12	6.42	0.1/wt.%
GFMK-7.5		7.5		
GFMK-3.75		3.75		
GFMK-12C	MK/GGBFS	12	20	
GFMK-7.5C		7.5		
GFMK-3.75C		3.75		
GFFA-75	FA	75	0.5	
GFFA-45		45		
GFFA-18		18		
GFFA-75C	FA/GGBFS	75	20	
GFFA-45C		45		
GFFA-18C		18		

## Data Availability

Data are available on request from the authors.

## References

[1] Singh R., Budarayavalasa S. (2021). Solidification and stabilization of hazardous wastes using geopolymers as sustainable binders. J. Mater. Cycles Waste Manag..

[2] Zifang X., Dongdong Y., Tao D., Yan D. (2021). Research on Preparation of Coal Waste-Based Geopolymer and Its Stabilization/Solidification of Heavy Metals. Integr. Ferroelectr..

[3] Luo Z., Zhi T., Liu L., Mi J., Zhang M., Tian C., Si Z., Liu X., Mu Y. (2022). Solidification/stabilization of chromium slag in red mud-based geopolymer. Constr. Build. Mater..

[4] Lan T., Meng Y., Ju T., Song M., Chen Z., Shen P., Du Y., Deng Y., Han S., Jiang J. (2022). Manufacture of alkali-activated and geopolymer hybrid binder (AGHB) by municipal waste incineration fly ash incorporating aluminosilicate supplementary cementitious materials (ASCM). Chemosphere.

[5] Sata V., Sathonsaowaphak A., Chindaprasirt P. (2012). Resistance of lignite bottom ash geopolymer mortar to sulfate and sulfuric acid attack. Cement. Concr. Comp..

[6] Liew Y.-M., Heah C.-Y., Mohd Mustafa A.B., Kamarudin H. (2016). Structure and properties of clay-based geopolymer cements: A review. Prog. Mater. Sci..

[7] Askarian M., Tao Z., Adam G., Samali B. (2018). Mechanical properties of ambient cured one-part hybrid OPC-geopolymer concrete. Constr. Build. Mater..

[8] Occhicone A., Graziuso S.G., De Gregorio E., Montagnaro F., Ricciotti L., Tarallo O., Roviello G., Ferone C. (2024). Synthesis and Characterization of New Acid-activated Red Mud-metakaolin Geopolymers and Comparison with their Alkaline Counterparts. J. Clean. Prod..

[9] El-Naggar M.R., Dong Y., Hamed M.M., El Abd A., Ibrahiem H.H., Gouda M.M., Mansy M.S., Hassan A.M.A., Rahman R.O.A. (2024). Microstructural Insights of Magnetic γ-Fe_2_O_3_/geopolymer Nanocomposite for Prospective Green Removal of Heavy Metals from Aqueous Solutions. Sep. Pur. Technol..

[10] El-Naggar M.R., El-Masry E.H., El-Sadek A.A. (2019). Assessment of Individual and Mixed Alkali Activated Binders for Solidification of a Nuclear Grade Organic Resin Loaded with 134Cs, 60Co and 152+154Eu Radionuclides. J. Haz. Mater..

[11] Ascensão G., Seabra M.P., Aguiar J.B., Labrincha J.A. (2017). Red Mud-based Geopolymers with Tailored Alkali Diffusion Properties and pH Buffering Ability. J. Cleaner Prod..

[12] Singh B., Ishwarya G., Gupta M., Bhattacharyya S. (2015). Geopolymer concrete: A review of some recent developments. Constr. Build. Mater..

[13] Kheimi M., Aziz I.H., Abdullah MM A.B., Almadani M., Abd Razak R. (2022). Waste Material via Geopolymerization for Heavy-Duty Application: A Review. Materials.

[14] Matsimbe J., Dinka M., Olukanni D., Musonda I. (2022). Geopolymer: A Systematic Review of Methodologies. Materials.

[15] Elyamany E.H., Elmoaty A.M.E.A., Elshaboury M.A. (2018). Setting time and 7-day strength of geopolymer mortar with various binders. Constr. Build. Mater..

[16] Nath S., Kumar S. (2020). Role of particle fineness on engineering properties and microstructure of fly ash derived geopolymer. Constr. Build. Mater..

[17] Chindaprasirt P., Chareerat T., Hatanaka S., Cao T. (2011). High-Strength Geopolymer Using Fine High-Calcium Fly Ash. J. Mater. Civ. Eng..

[18] El-Naggar R.M., El-Dessouky I.M. (2017). Re-use of waste glass in improving properties of metakaolin-based geopolymers: Mechanical and microstructure examinations. Constr. Build. Mater..

[19] Ouyang G., Wang J., Wang R., Chen L., Bu B. (2021). Rheokinetics and fluidity modification of alkali activated ultrafine metakaolin based geopolymers. Constr. Build. Mater..

[20] Huseien G.F., Mirza J., Ismail M., Hussin M.W. (2016). Influence of different curing temperatures and alkali activators on properties of GBFS geopolymer mortars containing fly ash and palm-oil fuel ash. Constr. Build. Mater..

[21] Nodehi M., Taghvaee M.V. (2021). Alkali-Activated Materials and Geopolymer: A Review of Common Precursors and Activators Addressing Circular Economy. Circ. Econ. Sust..

[22] Gao W., Zhou W., Lyu X., Liu X., Su H., Li C., Wang H. (2023). Comprehensive Utilization of Steel Slag: A review. Powder Technol..

[23] Sayenko S., Svitlychnyi Y., Shkuropatenko V., Pancotti F., Sandalova S., Poulesquen A., Giboire I., Hasnaoui A., Cori D., Magugliani G. (2024). Incorporation of Organic Liquid Waste in Alkali Activated Mixed Fly Ash/Blast Furnace Slag/Metakaolin-based Geopolymers. Nucl. Eng. Des..

[24] Yang Q.Z., Hou P.K., Guo T.T. (2011). Study on the Effects of Different Water-Cement Ratios on the Flow Pattern Properties of Cement Grouts. Appl. Mech. Mater..

[25] Peng H., Cui C., Cai C.S., Li S.L., Zhao J.W. (2016). Mechanism of activator concentration influencing properties of metakaolin-based geopolymer. Acta Mater. Compos. Sin..

[26] El-Naggar M., Amin M. (2018). Impact of alkali cations on properties of metakaolin and metakaolin/slag geopolymers: Microstructures in relation to sorption of 134 Cs radionuclide. J. Hazard. Mater..

[27] Yip C., Lukey G., Deventer V.J. (2004). The coexistence of geopolymeric gel and calcium silicate hydrate at the early stage of alkaline activation. Cem. Concr. Res..

[28] Wang A., Zhang C., Sun W. (2004). Fly ash effects: III. The microaggregate effect of fly ash. Cem. Concr. Res..

[29] Wang A., Zhang C., Sun W. (2004). Fly ash effects: II. The microaggregate effect of fly ash. Cem. Concr. Res..

[30] Sun Y., Hu S., Zhang P., Elmaadawy K., Ke Y., Li J., Li M., Hu J., Liu B., Yang J. (2020). Microwave enhanced solidification/stabilization of lead slag with fly ash based geopolymer. J. Cleaner Prod..

[31] National Library of Standards: GB/T 1346-2011. https://www.nssi.org.cn/nssi/front/77257743.html.

[32] National Library of Standards: GB/T 8077-2012. https://www.nssi.org.cn/nssi/front/124563566.html.

[33] Lin B.Q., Zhong Y.T., Cao X., Liu T., Wang Y.H. (2021). Effect of microwave irradiation on pore and fracture evolutions of coal. J. Xi’an Univ. Sci. Technol..

